# Expression of *Sox2* and *Oct4* and Their Clinical Significance in Human Non-Small-Cell Lung Cancer

**DOI:** 10.3390/ijms13067663

**Published:** 2012-06-21

**Authors:** Xinxin Li, Jinguang Wang, Zhiyun Xu, Aftab Ahmad, Encheng Li, Yuan Wang, Suli Qin, Qi Wang

**Affiliations:** 1 Department of Respiratory Medicine, The Second Hospital Affiliated to Dalian Medical University, Dalian 116023, China; E-Mails: lixinxin1212@hotmail.com (X.L.); tutuxzy@163.com (Z.X.); aftab_ahmad17@hotmail.com (A.A.); doctorliencheng@163.com (E.L.); wang.yuan21@hotmail.com (Y.W.); 2 Department of Thoracic Surgery, The First Hospital Affiliated to Dalian Medical University, No. 222 Zhongshan Road, Dalian 116011, China; E-Mail: dlwangjg@hotmail.com; 3 People’s Military Medical Press, Beijing Fuxing Road 22 A3, Beijing 100842, China; E-Mail: qinsuli@pmmp.com.cn

**Keywords:** NSCLC, *Sox2*, *Oct4*, immunohistochemistry, RT-PCR, Western blot

## Abstract

*Sox2* and *Oct4* are transcription factors with the characteristics of regulating self-renewal and differentiation of embryonic stem cell. The aim of this study was to detect the expression of *Sox2* and *Oct4* and analyze their clinical significance in human non-small-cell lung cancer (NSCLC). Expression of *Sox2* and *Oct4* were assayed in cancer tissues and their corresponding paracancerous tissues from 44 patients with NSCLC and 21 patients with benign tumors using immunohistochemistry, Western blot, reverse transcription polymerase chain reaction (RT-PCR). The correlation between the expression of *Sox2* and *Oct4* and tumor type, grade and prognosis and the utility of the two genes in discriminating between benign and malignant tumors were analyzed as well. The results showed that *Sox2* and *Oct4* positive staining was only seen in the nuclei of cancer cells but not in either the precancerous tissues or benign tumor tissues by immunohistochemistry (*p* < 0.01). Furthermore, in the lung cancer tissue, the positive rate for *Sox2* and *Oct4* was 70.5% and 54.5%, respectively. Meanwhile, clinicopathological correlations showed that the *Oct4* expression level was significantly associated with poorer differentiation and higher TNM stage of the cancer (*p* < 0.05). Western blot and RT-PCR analysis showed similar results to immunohistochemistry. Follow-up analysis revealed that expression of *Oct4* was significantly associated with poor prognosis of lung cancer. The conclusion is that *Sox2* and *Oct4* may act as the promising unit markers in directing NSCLC diagnosis and therapy. Also, *Oct4* can be regarded as a novel predictor of poor prognosis for NSCLC patients undergoing resection.

## 1. Introduction

Lung cancer is by far one of the most common malignant tumors worldwide. It is also the leading cancer type for cancer mortality that takes account for more than 26% of all cancer deaths [1]. This is largely due to the lack of biomarkers for early diagnosis. Since the common used methods such as X-ray and sputum cancer cell assay turn out only to be useful for the diagnosis for the late stage of lung cancer, while less effective for the earlier tumor stages, which directly weaken the effect of therapy and lead to a poor prognosis. Therefore, lung cancer biomarkers have been taking a relatively important place in early diagnosis, therapy guidance and predicting prognosis [2,3]. Unfortunately, the currently available lung cancer biomarkers such as CEA, NSE, Cyfra21-1, CA125 are not sensitive or specific enough to be effective detection means in diagnosis, drug responses, or prognosis [4]. In view of this, more novel and effective biomarkers need to be discovered and utilized. Cancer stem cells (CSCs) are considered as a small subpopulation of cancer cells that is highly enriched with the properties of self-renewal, extensive proliferation and tumor formation [5]. Substantial and growing experimental evidence is suggesting that many cancers may be driven by this subpopulation, and it can be used as a biomarker for the diagnosis of this type of cancer. More and more CSCs have been identified and isolated in solid cancers including ovarian cancer, neuroblastoma, breast cancer and lung cancer [6,7]. Kem *et al.* reported that bronchoalveolar stem cells (BASCs) have the properties of self-renewal, pluripotency and proliferation, and suggested them as progenitors for lung adenocarcinoma [8].

There are many members of CSCs. *Sox2* is an important member of the sox gene family. The sox genes encode transcription factors that interact with DNA through their highly conserved high mobility group (HMG) domain [9,10]. It is known that sox genes are expressed in a wide variety of tissues and play important roles in the regulation of organ development and cell type specification, especially in embryonic stem cell (ESC) development [11,12]. *Sox2* is also regarded as a key factor for some cancer progress [13]. *Oct4* (also known as Octamer 4) belongs to the family of POU-domain transcription factors and was first found in embryonic stem (ES) and germ cells [14]. Many studies indicated that *Oct4* plays a pivotal role in maintaining the self-renewal and pluripotency of ES cells [15–17]. More recently, a growing number of cancer cells were confirmed to express *Oct4*, even Monk *et al.* found that the gene was expressed only in tumors, but not in normal somatic cells, which demonstrated that *Oct4* may also be crucial in cancer development [18]. Either in ES cells or in CSCs, *Sox2*, together with *Oct4*, plays an important role in regulating stem cell characteristics [17,19]. The two genes constitute part of an important gene regulatory network and are essential for embryogenesis and the pluripotency and self-renewal of the cells. Many recent studies found that some cancers including breast carcinomas, germinomas, and pancreatic carcinomas expressed *Sox2* and *Oct4* simultaneously [20–22] and their expression was associated with the differentiation of the tumors [23]. All this means that the two genes appear to be significant for cancer cells’ survival. For lung cancer, however, very rare reports have involved the two genes till now. In this study, we used immunohistochemistry (IHC), Western blot, and reverse transcription polymerase chain reaction (RT-PCR) to assay the expression of *Sox2* and *Oct4* in 44 non-small-cell lung cancer (NSCLC) mainly including squamous cell carcinoma (SCC) and adenocarcinoma patients and 21 benign pulmonary tumor patients. The correlation between the expression of *Sox2* and *Oct4* and tumor type, grade, prognosis and the utility of the two genes in discriminating between benign and malignant tumors was analyzed as well.

## 2. Results

### 2.1. Sox2 and Oct4 Expressions in Cancerous Tissues, Precancerous Tissues and Lung Benign Tumor Tissue

**Figure 1:**
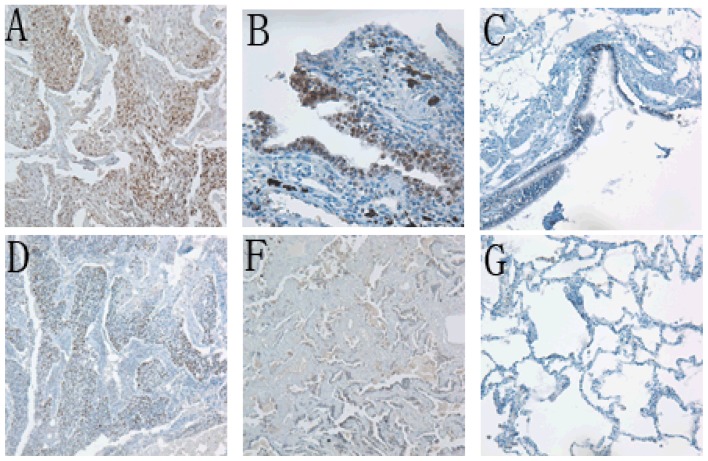
Representative expression patterns of *Sox2* (**A**–**C**) and *Oct4* (**D**–**F**) in cancerous tissues and their corresponding paracancerous tissues. Brown grains represent a positive signal. The positive expression site of *Sox2* and *Oct4* was mainly localized in the nucleus of tumor cells. *Sox2* positive expression in lung squamous cell carcinoma (SCC) (**A**) and lung adenocarcinoma (**B**); *Sox2* expression negative in paracancerous tissue (**C**); *Oct4* staining positive in lung SCC (**D**) and lung adenocarcinoma (**E**); *Oct4* negative expression in paracancerous tissue (**F**).

We investigated *Sox2* and *Oct4* protein expression by IHC in 44 human NSCLC cancerous and precancerous tissues and 21 human benign tumor tissues. Overall, for both *Sox2* and *Oct4*, only immunostaining in the nuclei of the cancer cells was thought to be positive, whereas all the precancerous tissues and benign tumor tissues showed negative staining with significant differences (*p* < 0.01, Table 1, Figure 1). Among the 44 cancerous tissues, 31 showed positive staining for *Sox2* with a positive rate of 70.5%, and 24 showed positive staining for *Oct4* with a positive rate of 54.5%. Meanwhile, 16 cases showed both *Sox2* and *Oct4* positive staining, with a co-positive rate of 36.4% (16/44); and 39 cases showed either *Sox2* or *Oct4* positive staining, with a co-positive rate as high as 88.6%.

**Figure 2:**
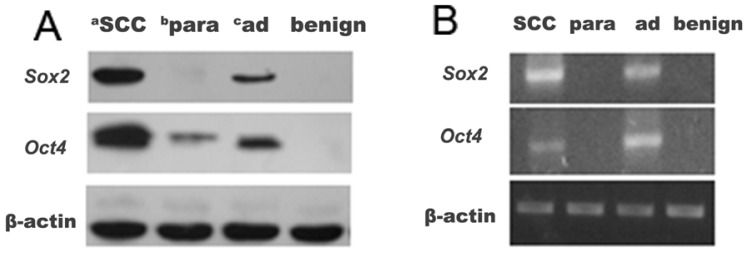
Western blot and reverse transcription polymerase chain reaction (RT-PCR) analysis on the expression of *Sox2* and *Oct4* in lung SCC, adenocarcinoma, paracancerous and benign tumor tissues; (**A**) Western blot; (**B**) RT-PCR. ^a^ SCC = squamous cell carcinoma; ^b^ para = precancerous; ^c^ ad = adenocarcinoma.

In order to confirm the IHC results, Western blot and RT-PCR were performed. On the whole, both the Western blot and RT-PCR analysis results were in line with the IHC results. That is the expression of the two factors of *Sox2* and *Oct4* in NSCLC tissues was significantly higher than that of their paracancerous tissues and the benign tumors at both the protein and mRNA level (Figure 2). Nevertheless, small differences were still seen. For example, some cases with negative expression of *Sox2* and *Oct4* by IHC contrarily showed low-expression of them in Western blotting or RT-PCR, as shown in Figure 2. This may be due to the rough judgment of IHC.

### 2.2. Clinicopathological Correlations

The association between *Sox2* and *Oct4* expression levels and the clinicopathological characteristics of the lung cancer patients is summarized in Table 2. Neither *Sox2* nor *Oct4* expression was correlated to age, sex, tumor location and pathological type. However, higher levels of *Oct4* expression were significantly associated with poorer differentiation and higher TNM stage of the cancer (*p* < 0.05), whereas *Sox2* did not have this correlation.

### 2.3. Correlation between Sox2 and Oct4 Expression

These two transcription factor expressions can be observed co-localized in the same areas of the cell nuclei in some malignant tumors with around 36.4% of the cases showing both *Sox2* and *Oct4* positive staining and around 88.6% of the cases showing either *Sox2* or *Oct4* positive staining, indicating that the joint detection of the two factors is helpful. However, their expressions were also independently heterogeneous: High expression of *Sox2* (grade 3) always lead to low expression (grade 2) or even negative detection of *Oct4* (data not shown). In order to know whether the two factors *Sox2* and *Oct4* correlated statistically, a cross analysis was performed, and the results showed that their expressions were not significantly correlated (*p* > 0.05, Table 3).

### 2.4. Relation to Survival

**Figure 3:**
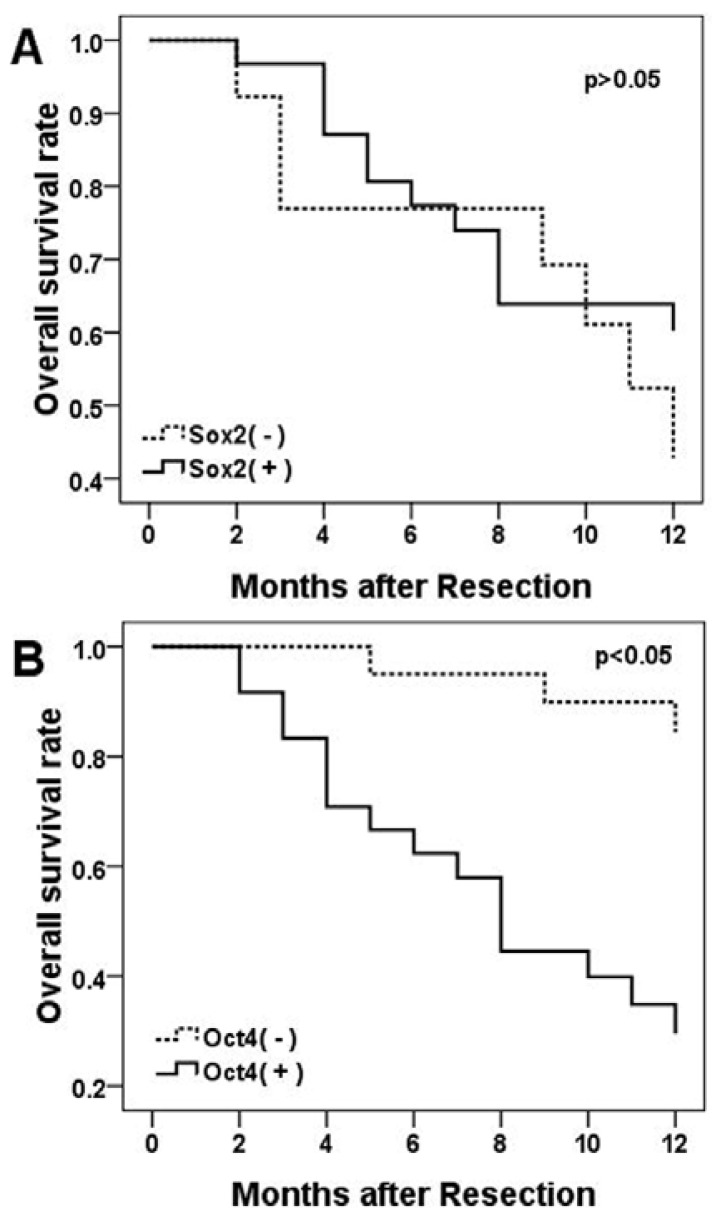
Kaplan-Meier curves for overall survival rates according to *Sox2* expression status (**A**) and *Oct4* expression status (**B**). Positive expression of *Oct4* was significantly associated with poor overall survival (*p* < 0.05).

Overall survival was measured for the NSCLC patients from the onset of treatment to the date of death or the survival status at the last date of the one-year follow-up. During the one-year follow-up period, 16 patients were found with recurrence or metastases, 19 patients died of cancer-related causes. A multivariate survival analysis was performed with a Cox regression model for each predictor of prognosis of IHC results. The results showed that patients with positive *Oct4* immunostains had an overall poorer survival compared to the patients with negative stained tumors (*p* = 0.008, Figure 3). However, there was no significant correlation between *Sox2* immunostaining positive and survival for the NSCLC patients (*p* > 0.05, Figure 3).

## 3. Discussion

In this study, we investigated the expression of *Sox2* and *Oct4* in 44 human NSCLC cancerous and their precancerous tissues and 21 benign human tumor tissues. The present investigation revealed several novel observations. First, the results showed that there were 70.5% and 54.5% positive expressions of *Sox2* and *Oct4*, respectively, for NSCLC. However, all of their corresponding paracancerous tissues and the benign tumor tissues were negative for these two transcription factors. All this indicates that *Sox2* and *Oct4* can be potential novel biomarkers to distinguish cancer from noncancerous lesions and benign lung tumors. Second, *Oct4* but not *Sox2* expression level was significantly associated with poorer differentiation and higher TNM stage of the tumors and poor clinical outcome of the patients (*p* < 0.05). Moreover, neither *Sox2* nor *Oct4* expression was correlated to age, sex, tumor location, or histological type of the cancers. Third, each of *Sox2* and *Oct4* alone had some limitation of sensitivity as a biomarker of NSCLC, however, when combined, the sensitivity was significantly improved to 88.6%, which suggests that *Sox2* and *Oct4* can be regarded as coalition predictive factors for patients with lung adenocarcinoma or SCC, and the significance of co-detection of the two factors was much more sensitive than detection of one factor alone.

Since the understanding of lung cancer tumor genesis and development may provide the possibility of its therapy or even prevention, the CSCs theory might be one of the ideal models to achieve this aim. As crucial transcription factors that maintain embryonic stem cell differentiation and pluripotency, *Sox2* and *Oct4* have also been recognized as having “stemness” characteristics in cancer cells in more recent research, therefore, *Sox2* and *Oct4* may lead to cells immortality, self-renewable and invasive properties of cancer cells [13–19]. Supporting this hypothesis, it was suggested that knocking down *Sox2* and *Oct4* in tumor-initiating cells would lead to the loss of the self-renewal, proliferating and tumorigenic capacities and result in CSC-like cell apoptosis of cancer cells [24,25].

Adenocarcinoma and SCC are the most frequent and aggressive lung cancer types of NSCLC and previous studies have pointed out that stem cells must be involved in their development and maintenance. Bronchoalveolar stem cells (BASCs), which were capable of multipotent differentiation and self-renewal in both normal lung and lung cancer, are involved in the process normal cells of the distal lung developing into adenocarcinoma [26]. In other words, BASCs can be seen as the putative cells of origin for this subtype of lung cancer. Some reports shown that *Oct4* is exhibited in BASCs [8]. Our study suggests that not only *Oct4* but also *Sox2* may play an impotent role in this procedure of lung cancer tumorigenesis.

For SCC, the cancer came from bronchial epithelium and commonly arose near a central bronchus. Loss of the 3p arm and amplification of the 3q25–27 region were identified in most of the NSCLC cases, these genomic imbalances seem to contribute to the pathogenesis of lung cancer, especially the product of 3q26 is potentially involved in the control of cell proliferation and malignant transformation [27]. Further investigations have indicated that 3q26.3 aberrations, in which *Sox2* acts as a driver gene, were proposed as an oncogene in lung SCC and *Sox2* actions along the carcinomagenesis sequence [28–30]. Our work supported that *Sox2* might be a promising target for lung SCC therapy, as well as a director to lung SCC. And because of the absence of *Sox2* in benign lung tumors and normal lung tissues, this target therapy might have less side effects. Whether *Oct4* plays a similar role needs to be studied further. For adenocarcinoma, no relevant studies have been reported yet, but our research revealed no significant difference for the expression of *Sox2* and *Oct4* between SCC and adenocarcinoma. So suggest the hypothesis that the two transcription factors play an important role in the adenocarcinoma neoplastic processes as well. More research needs to be carried out to clarify the mechanism.

Although *Sox2* and *Oct4* expressions performed independently in some of the cases, they were more often co-located in the same area. Possible linkages between them in lung cancer are considered, such as the *Sox2*-*Oct4* complex. It was revealed that they co-occupy the promoters of several hundred target genes and also form core transcription regulatory circuitry together [19]. Several studies have suggested that the *Sox2*-*Oct4* complex is essential for the transforming growth factor-β (TGF-β) and wnt/β-catenin pathways, which have emerged as other important players in the self-renewal and maintenance of cancer stem cells [31].

Interestingly, our results showed that only 36.4% of all samples co-expressed *Sox2* and *Oct4* and that their expression levels were separate, or even reversed. This finding raised a new puzzling question: What is the relationship between *Sox2* and *Oct4*, cooperative or interactive? In human ESCs, *Sox2* and *Oct4* are thought to be regulated by autoregulatory loops, and over-expression of *Sox2* caused a decrease in *Oct4* [31,32]. Bernadt *et al.* demonstrated that over-expression of *Sox2*, but not *Oct4*, inhibited the activity of *Oct4* promoter in ES cells [33]. Although this finding was mainly observed in ES cells, lung SCC appears to be the likely trend in our work: The *Sox2* over-expression always lead to *Oct4* under-expression or non-expression. We have reasonable ground to propose that in lung SCC, the *Sox2* and *Oct4* regulation network also works to co-effect the cancer stem cell differentiated and self-renewal. However, the mechanisms involved are far from clear, and more work should be performed. What we confirmed is that the combinatorial analysis of *Sox2* and *Oct4* expression in NSCLCs is more helpful than the single analysis.

The current results demonstrate that *Sox2* and *Oct4* can be detected in human lung cancer by IHC, Western blot and RT-PCR. However, there are some differences among them that can hardly be ignored, for example, some cases display expression of the two factors with Western blot and RT-PCR, while they cannot be observed when staining with IHC in cancer cell. The reason might be that *Sox2* and *Oct4* mainly express in cancer cells, but a small amount of other cells such as leukomonocytes or interstitial cells can also express these and hence be detected with these tests. It also may be due to the rough judgment of IHC. However, IHC is more visualized and veracious for clinical samples.

Other studies have also shown that *Sox2* or *Oct4* over-expression is associated with poor prognosis in diverse cancers [13,34]. However, to our knowledge, there is little information about the prognostic value of the two factors in human NSCLC. Our research found that a positive expression of *Oct4* protein and mRNA were independent prognostic factors for a poor overall survival of lung cancer patients.

The present study indicated that *Oct4*, but not *Sox2*, was a novel marker in predicting the prognosis of NSCLC. To further confirm the significance of the two factors in the diagnosis and therapy of NSCLC, our group is now working on an easy-test of *Sox2* and *Oct4* and a quick knocking down of the two transcription factors.

## 4. Experimental Section

### 4.1. Specimens and Clinical Pathological Data

This study was approved by the Institutional Review Board and Human Ethic of the First affiliated hospital of Dalian Medical University and the Second affiliated hospital of Dalian Medical University. Forty-four NSCLC patients and 21 pulmonary benign tumor patients who underwent surgical treatment in the First or the Second affiliated hospital of Dalian Medical University during the period from 2008 to 2011 were collected. All the patients were diagnosed with pathologic identification. Among them, 45 were men and 20 were women, aging from 37 to 78 years of age with a mean age of 62.6. The 44 lung cancers included 23 adenocarcinoma and 21 SCCs; 21 benign tumor included six tuberculosis and 15 inflammatory disease cases. The describing data of the 65 patients are listed in Table 2. Surgically removed specimens of fresh lung cancer tissues, their paracancerous tissues and benign tumor tissues were partly fixed in buffered formalin and embedded in paraffin blocks for IHC analysis and the rest of the samples were stored in the refrigerator at −80 °C for RT-PCR and Western blot.

### 4.2. Imunohistochemical Analysis

Immunohistochemical stains were performed on formalin-fixed, paraffin-embedded 5 μm sections using the SP-9000 Histostain tm-plus kits (ZYMED, USA). The sections were deparaffinized in xyliene, and microwaved in antigen retrieval buffer for 15 min. Endogenous peroxidase activity was blocked by incubation in 3% H_2_O_2_ solution in methanol for 10 min. Slides were blocked in 10% goat serum in TBS for 15 min, and then monoclonal mouse anti-Sox2 or anti-*Oct4* antibody (Sigma, USA) both diluted at 1:100 was added for incubation for 4 °C overnight. Specimens were stained with 3′,3-diaminobenzidine tetrahydrochloride (DAB), counterstained with hematoxylin, dehydrated and mounted in Diatex. A PBS-only staining sample was used as a background control, gastric cancer and seminoma tissues were used as positive controls for *Sox2* and *Oct4*, respectively.

Only nuclear staining was considered as positive. *Sox2* and *Oct4* expression were defined as any of the following four patterns and scored according to the extent of positivity of the stained cells: 0, no positive cells seen; 1, around <10% of cells stained; 2, around 10%–50% of cells stained; and 3, around >50% of cells stained; for statistical analyses, the grades 0 and 1 were considered as negative, the grades 2 and 3 were considered as positive. The details were described in our previous work [13].

### 4.3. RT-PCR

Total RNA was isolated from the fresh tissue samples of lung cancer tissues, their paracancerous tissues and benign tumor tissues using Trizol reagent (Takara, China) according to the manufacturer’s protocol. Extracted RNA was dissolved in RNA free water. cDNA was synthesized with the RNA PCR kit (Takara, China). RT-PCR for *Sox2*, *Oct4* and β-actin was performed using the primer sequences given in Table 4. PCR products were analyzed by electrophoresis of 10 μL of each PCR reaction mixture in a 1.5% agarose gel.

### 4.4. Western Blot Analysis

Proteins isolated from the fresh tissue samples of lung cancerous tissues, their paracancerous tissues and benign tumor tissues using PARA kit (Beyotime, China) according to the manufacturer’s protocol. 10 μL of the protein samples were separated on a 10% SDS-PAGE gel (Bio-Rad) for 120 min at 200 mA, and then transferred to nitrocellulose membrane (Whatman, Kent, UK). After blocking in 5% fat-free milk, the membrane was incubated with the dilution of the anti-*Sox2* antibody (1:100) and anti-*Oct4* antidody (1:100), respectively, overnight at 4 °C and the dilution of the secondary IgG-horseradish peroxidase (HRP) conjugated antibody for 1 h at room temperature. The stained membranes were visualized by enhanced chemiluminescence reaction using the ECL Plus (GE Healthcare, USA).

### 4.5. Follow-Up

Follow-up information was available for the NSCLC patients for a period of minimum 1 year. All patients were monitored prospectively by physical examination, X-ray or ultrasonography every 1–3 months thereafter for surveillance of recurrence or metastases. The recurrence, metastases or death times were recorded.

### 4.6. Statistical Analysis

SPSS 17.0 was employed to analyze all data obtained in this study. The pearson chi-square was used for positive staining rates comparison between subgroups. The bands of Western blot and RT-PCR were evaluated using the *t*-test. A multivariate survival analysis was performed with a Cox regression model for each predictor of prognosis, as well as 95% confidence intervals. Overall survival was assessed by Kaplan-Meier analysis. Only *p* < 0.05 was considered as statistical significance.

## 5. Conclusions

In summary, transcription factors *Sox2* and *Oct4* were expressed and localized in the nucleus of the wide majority of lung SCC and adenocarcinoma, but not in their paracancerous tissues and benign tumor tissues, suggesting that *Sox2* and *Oct4* can act as novel unite markers and ideal therapeutic targets. The expression of *Oct4* enables the tumors to have a higher degree of stemness tumor cells, which in turn results in poorer clinical outcome for patients with NSCLCs.

## Figures and Tables

**Table 1:** *Sox2* and *Oct4* expressions in cancerous tissues, precancerous tissues and benign lung tumor tissue.

	Cancerous tissues			Paracancerous tissues			Benign tumor tissues		
			
	a Pos	b Neg	Totle	Pos	Neg	Totle	Pos	Neg	Totle
*Sox2*	31 (70.5%)	13	44	0	44	44	0	21	21
*Oct4*	24 (54.5%)	20	44	0	44	44	0	21	21

a Pos = positive;

b Neg = negative.

**Table 2:** Correlation of *Sox2* and *Oct4* expressions to clinicopathological features of non-small-cell lung cancer (NSCLCs).

Variables	Cases	*Sox2*			*Oct4*		
	
		a Neg	b Pos	*p*	Neg	Pos	*p*
Age (year)				0.831			0.614
<60	18	5	13		9	9	
≥60	26	8	18		11	15	
Gender				0.340			0.757
Male	33	11	22		15	17	
Female	11	2	9		5	7	
Location				0.469			0.507
Right	24	6	18		12	12	
Left	20	7	13		8	12	
Differentiation				0.755			0.009 *
Well	14	5	9		10	4	
Moderately	13	4	9		7	6	
Poorly	17	4	13		3	14	
TNM stage				0.318			0.015 *
I~II	29	10	19		17	12	
III~IV	15	3	12		3	12	
Pathological type				0.599			0.378
Adnocarcinoma	23	6	17		9	14	
SCC	21	7	14		11	10	

* *p* < 0.05;

a Neg = negative;

b Pos = positive.

**Table 3:** *Sox2* and *Oct4* expression cross tabulation.

	*Oct4*		Total
		
*Sox2*	negative	positive	
**negative**	5	8	13
**positive**	15	16	31
**Total**	20	24	44

**Table 4:** Primers for *Sox2*, *Oct4* and β-actin genes.

Gene name	Primer sequence	Size (bp)	Annealing temperature (°C)
*Sox2*	F cccccggcggcaatagca R tcggcgccggggagatacat	448	58
*Oct4*	F tcccttcgca agccctcat R tgacggtgcagggctccggggaggccc catc	408	55
β-actin	F gatcttgatcttcattgtgctggg R tcgtcaccaactgggacgacatgg	752	55
